# Supplementary material for: Phosphorylation in the Charged Linker Modulates Interactions and Secretion of Hsp90β
Source: Cells. 2021 Jul 5;10(7):1701. doi: 10.3390/cells10071701 (PMC8304327; doi:10.3390/cells10071701)
Supplement: Supplementary file 1 [file cells-10-01701-s001.zip › Supplementary/SupplementaryMaterial.pdf]

Supplementary material and figures

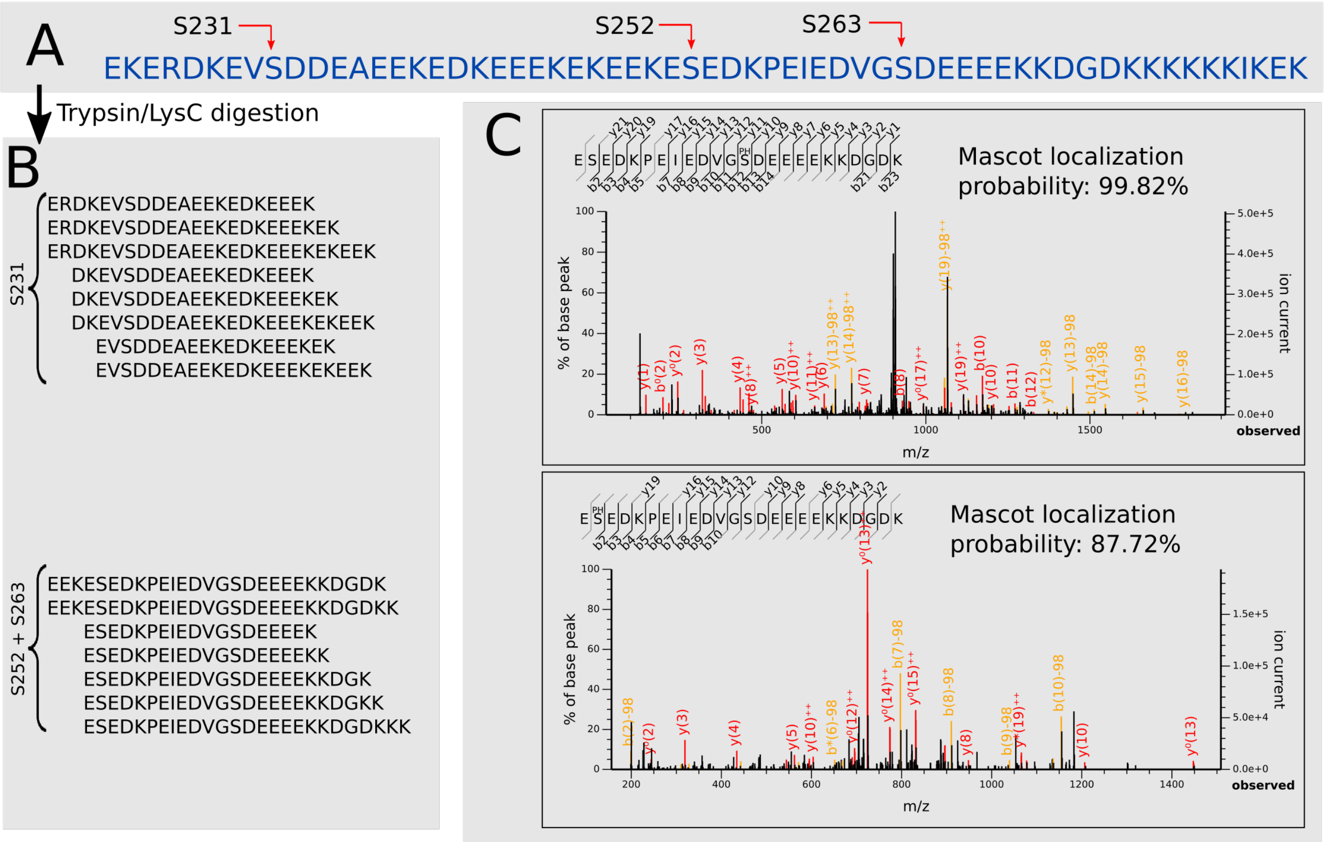

D	K562	AsPC-1	MDA-MB-468
ERDKEVSDDEAEEKEDKEEEK	NA	96.9%	94.9%
ERDKEVSDDEAEEKEDKEEEKEKEEK	95.2%	96.6%	94.4%
DKEVSDDEAEEKEDKEEEK	97.0%	97.0%	95.3%
DKEVSDDEAEEKEDKEEEKEK	96.4%	96.8%	94.9%
DKEVSDDEAEEKEDKEEEKEKEEK	96.4%	96.9%	94.4%

Supplementary figure S1. Determination of occupancy of S231 phosphosite in human Hsp90α. **A** Amino acid sequence of human Hsp90α with S231, S252, and S263 indicated by red arrows. **B** Various detected peptides spanning S231/S252/S263 resulting from tryptic digestion. **C** MS/MS spectra for peptide ESEDKPEIEDVGSDEEEEKKDGDK with phospho group on S252 (lower spectrum) and on S263 (upper spectrum) and localization probability. **D** Phosphorylation occupancy of S231 in Hsp90α in K562, AsPC-1, and MDA-MB-468 cells determined using different peptides.

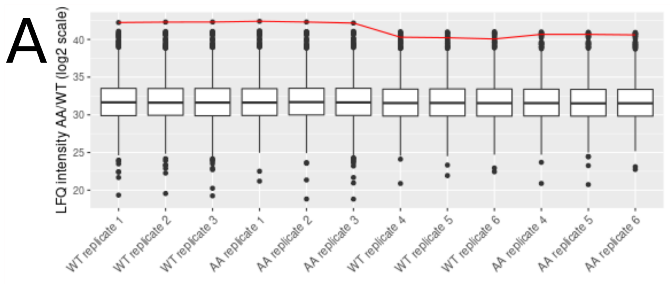

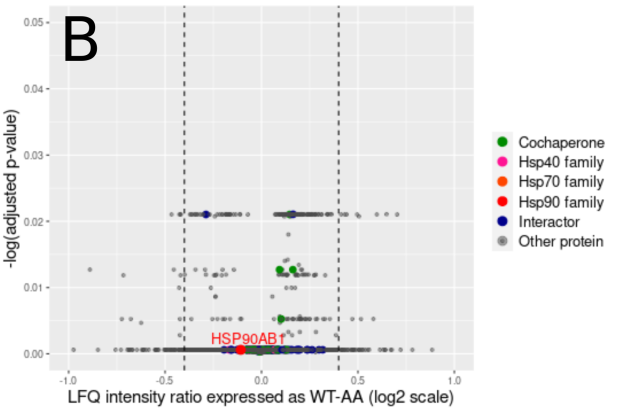

C

	Replicate 1	Replicate 2	Replicate 3
**EKEISDDEAEEEK**	97.1%	96.7%	97.5%
**EISDDEAEEEK**	93.8%	96.6%	96.0%
**IEDVGSDEEDDSGK**	89.1%	96.4%	94.0%
**IEDVGSDEEDDSGKDKK**	95.9%	97.5%	96.6%

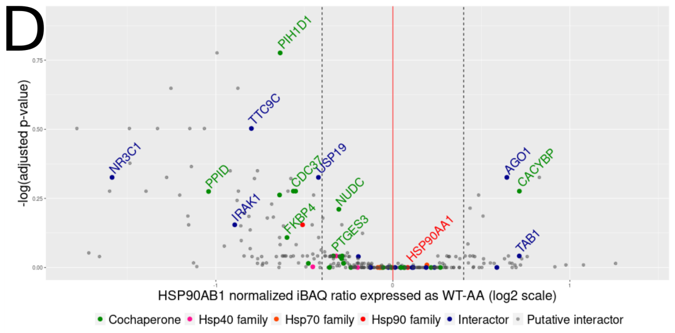

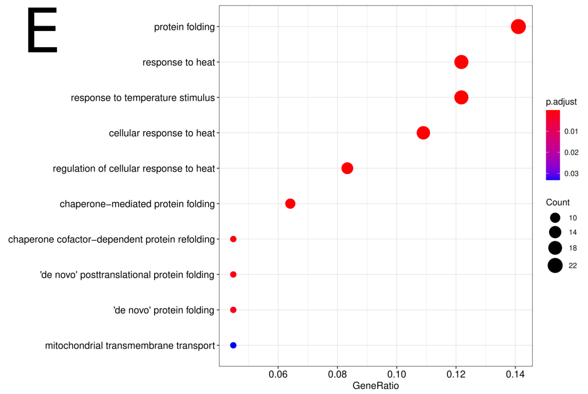

Supplementary figure S2. Label-free quantitative exploration of the interactome of WT vs. AA mutant. **A** LFQ intensities for the input (cell extract) proteins and HA-Hsp90β (red line) represented as boxplots. **B** Volcano plot for the input proteins and HA-Hsp90β. **C** S226 and S255 phosphorylation occupancy for 3 HA-Hsp90β^{WT} transfection replicates. **D** Volcano plot of the proteins identified as specific interactors. The fold-change is derived from the iBAQ values normalized on HA-Hsp90β; positive fold-change means more abundant in the double mutant co-IP. Vertical dashed lines mark absolute fold-change values of 0.4. **E** GO term over-representation analysis of the proteins with increased binding to AA in the label-free interactome experiment. Vertical axis: enriched GO terms. Horizontal axis: the GeneRatio is the ratio between the number of proteins enriched with the corresponding GO term and the total number of proteins identified in the input assigned to the corresponding GO term. Spot size is proportional to the count of proteins enriched (legend on the right of the plot). Spot color corresponds to the adjusted p-value as described in the color scale on the right of the plot.

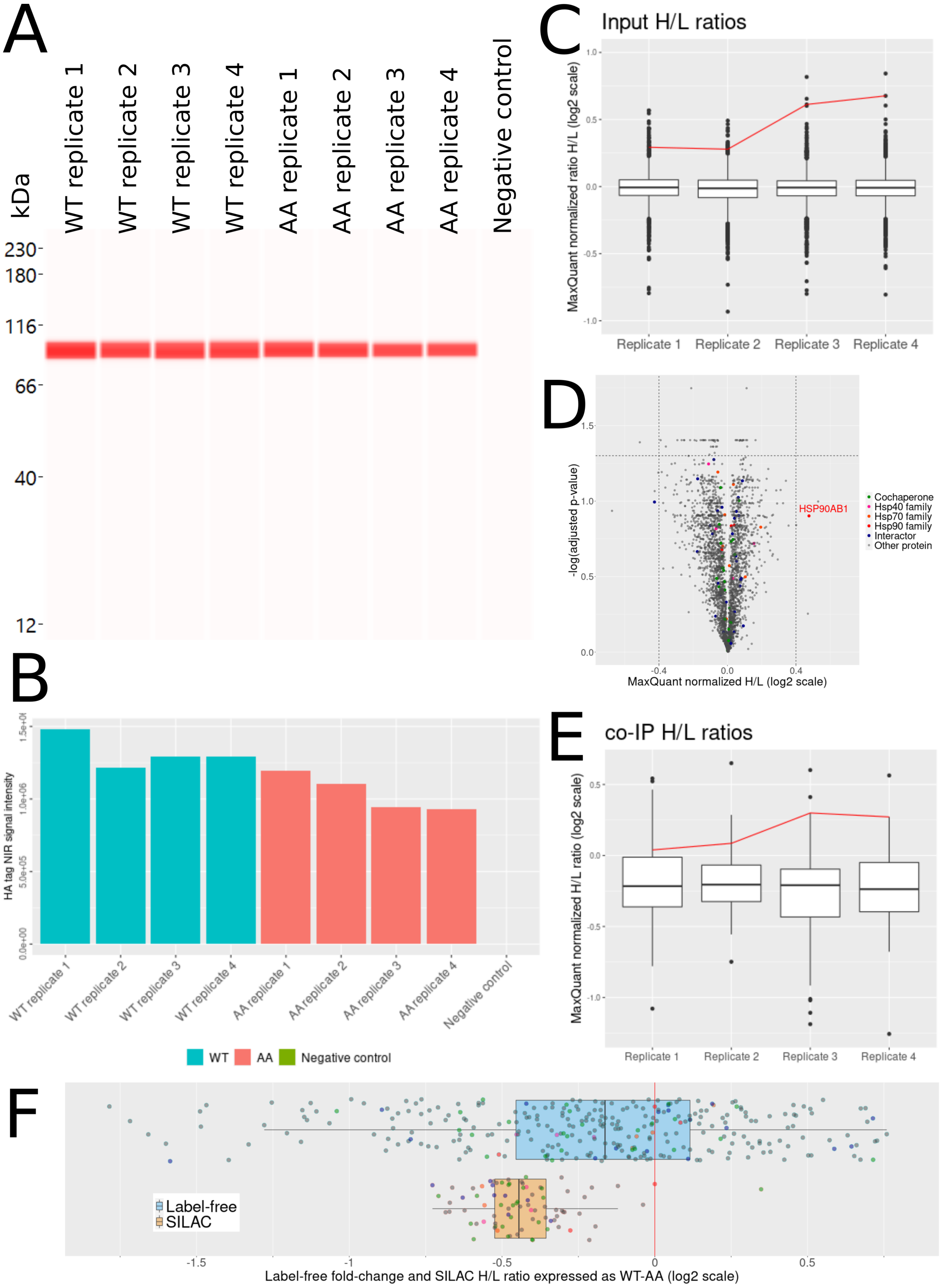

Supplementary figure S3. Analysis of transfected protein expression, input cell extracts, and co-IPs in the SILAC interactome experiment. **A** Capillary western blot analysis of HA-tag in the transfected samples. **B** Capillary western blot band intensities of panel A represented as bars. **C** MaxQuant normalized H/L ratios of the input lysates; Hsp90β marked by red line. WT is the heavy (H) sample and AA is the light (L) one. **D** Volcano plot of input lysates. **E** MaxQuant normalized H/L ratios of the proteins identified in the co-IPs and validated as specific interactor; Hsp90β marked by red line. **F** Boxplots of the Hsp90β normalized fold-changes in the label-free experiments and the Hsp90β normalized H/L ratios in the SILAC experiment (n=4). Hsp90β is indicated by the red line. Each point represents the average for a protein, color coded by class: cochaperones in green, Hsp40 proteins in pink, Hsp70 in orange, Hsp90 in red, other already known interactors in blue, and putative interactors in grey. Classification of cochaperones and known interactors is based on the “Hsp90 interactors” table by Didier Picard (www.picard.ch).

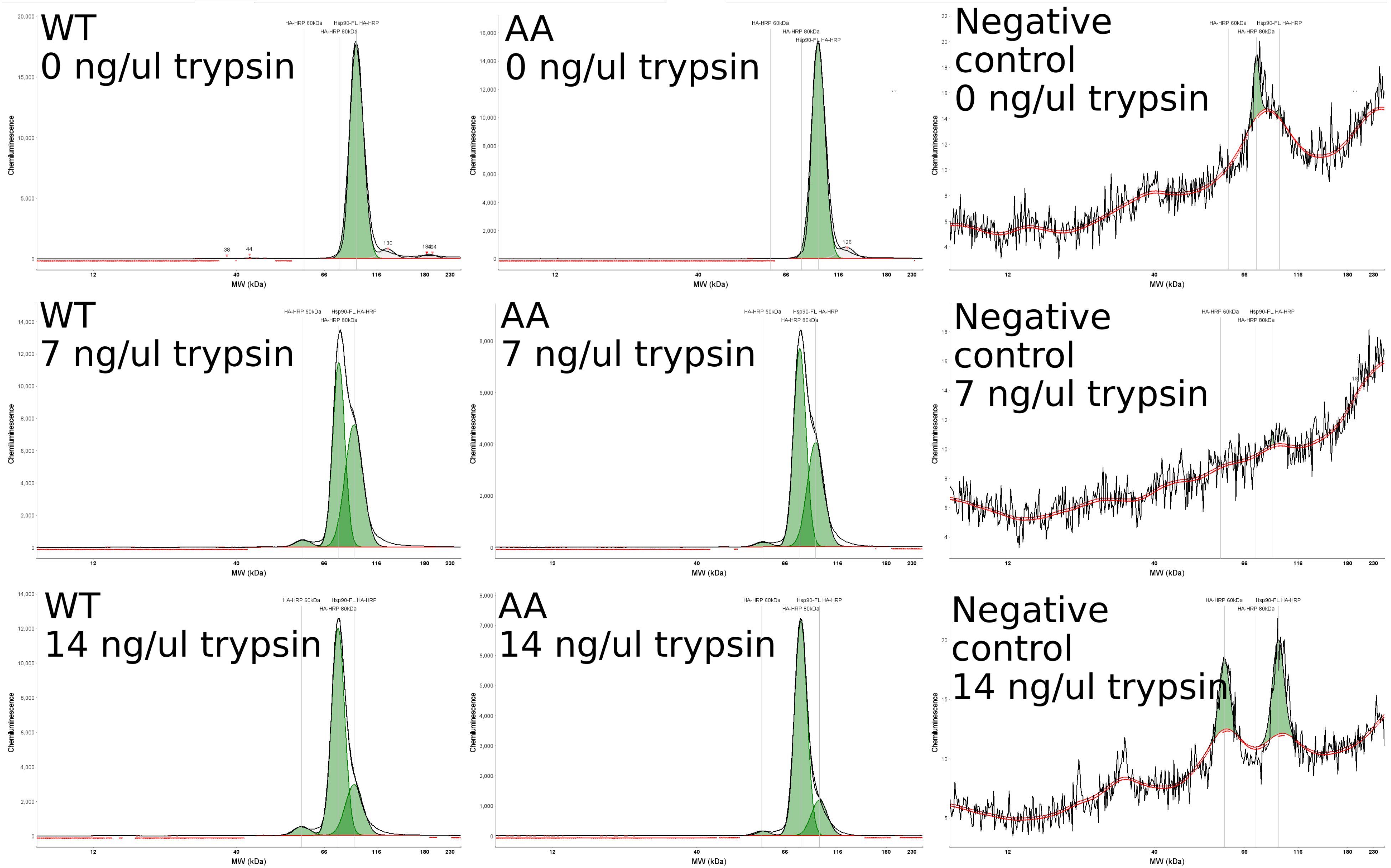

Supplementary figure S4. Graph view of capillary western blot detection of HA-tag for WT or AA Hsp90β for increasing trypsin concentration. The black trace is the measured chemiluminescence, the red trace is the baseline, and the green shaded areas the fitted peaks, indicated by the labels "HA-HRP 60kDa", "HA-HRP 80kDa", and "Hsp90-FL HA-HRP", corresponding respectively to a 60kDa cleavage product, an 80kDa cleavage product, and full-length HA-Hsp90.

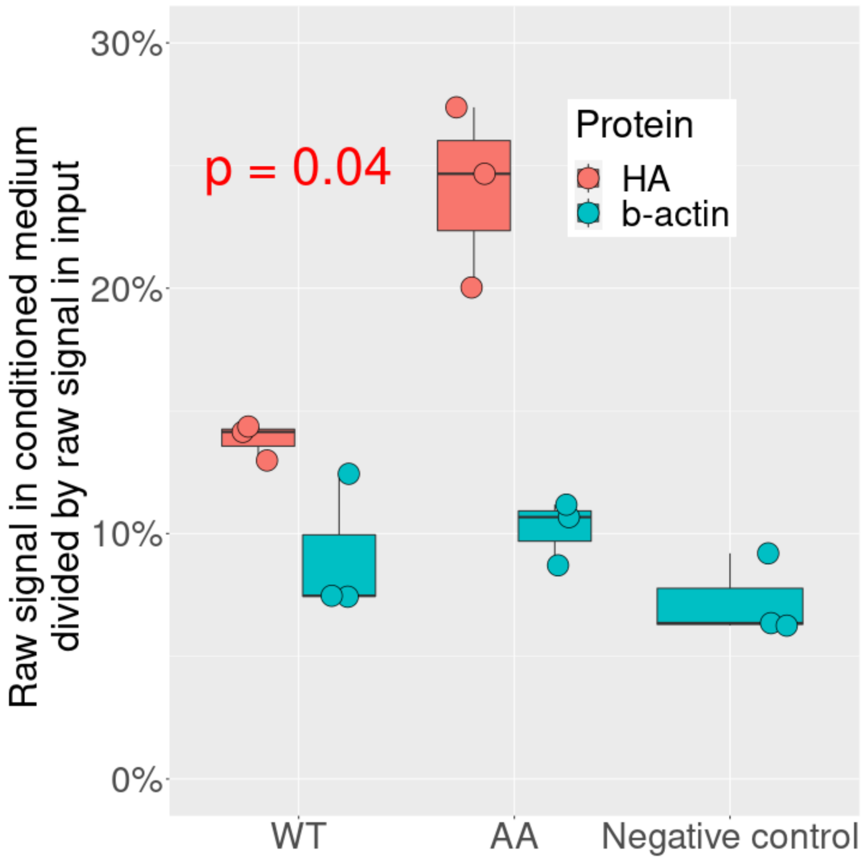

Supplementary Figure S5. Boxplots of the raw CWB signal in the conditioned medium of K562 cells divided by the raw CWB signal in input. Points represent replicates. The p-value indicated in red is from a t-test comparing HA-WT and HA-AA values. HA signal is in red and β-actin signal in blue.
